# Molecular Screening of Carbapenem-Resistant *K. pneumoniae* (CRKP) Clinical Isolates for Concomitant Occurrence of *Beta-Lactam* Genes (CTX-M, TEM, and SHV) in the Kingdom of Bahrain

**DOI:** 10.3390/jcm12247522

**Published:** 2023-12-05

**Authors:** Mohammad Shahid, Nermin Kamal Saeed, Nayeem Ahmad, Mohd Shadab, Ronni Mol Joji, Ali Al-Mahmeed, Khalid M. Bindayna, Khaled Saeed Tabbara, Abdulrahman Y. Ismaeel, Fazal K. Dar

**Affiliations:** 1 Department of Microbiology, Immunology, and Infectious Diseases, College of Medicine & Medical Sciences, Arabian Gulf University, Manama 329, Bahrain; nayeemahmad@agu.edu.bh (N.A.); shadab@agu.edu.bh (M.S.); ronnimj@agu.edu.bh (R.M.J.); aliem@agu.edu.bh (A.A.-M.); bindayna@agu.edu.bh (K.M.B.); khaledst@agu.edu.bh (K.S.T.); ismaeel@agu.edu.bh (A.Y.I.); fazalkd@agu.edu.bh (F.K.D.); 2 Microbiology Section, Department of Pathology, Salmaniya Medical Complex, Manama 435, Bahrain; nkamalh@hotmail.com

**Keywords:** carbapenem-resistant *K. pneumoniae*, *bla* genes, SHV, CTX-M-15, TEM, ERIC–PCR

## Abstract

The emergence of extended-spectrum β-lactamase-producing *Klebsiella pneumoniae*, including CRKP infections, has resulted in significant morbidity and mortality worldwide. We aimed to explore the presence of *bla* genes (CTX-M, TEM, and SHV) in CRKP isolates. A total of 24 CRKP isolates were randomly selected from the Salmaniya Medical Complex Microbiology Laboratory. These isolates, which were positive for carbapenemases, were further explored for CTX-M, TEM, and SHV genes using PCR. All the CTX-M PCR amplicons were sent for sequencing. To determine genetic relatedness, molecular typing by ERIC-PCR was performed. The *bla* gene testing demonstrated that a significant proportion of these isolates harbored SHV, CTX-M, and TEM genes (100%, 91.6%, and 45.8%), respectively. Bioinformatic analyses confirmed CTX-M-15 in these isolates. ERIC-PCR analysis showed three clusters demonstrating genetic relatedness. The study findings reveal the concomitant carriage of the SHV and CTX-M-15 and a comparatively lower carriage of TEM genes in CRKP isolates. Our findings highlight the significance of routinely reporting the presence of antibiotic resistance genes along with regular antibiotic sensitivity reports, as this will aid clinicians in prescribing appropriate antibiotics.

## 1. Introduction

The public health threat posed by multidrug-resistant (MDR) bacterial infections has grown globally. *Klebsiella pneumoniae* (*KP*), one of the most prevalent members of Enterobacterales causing community- and hospital-acquired illnesses, tends to be resistant to multiple antibiotics [1]. The synthesis of beta-lactamases is the most typical mechanism of beta-lactam antibiotic resistance. Extended-spectrum beta-lactamases (ESBLs) are to blame for the rise in resistance in modern medicine. They are plasmid-encoded enzymes that are inhibited by clavulanic acid and have the ability to hydrolyze bonds of β-lactam rings from antibiotics like penicillins, cephalosporins, and aztreonam [2]. Presently, more than 600 ESBL variations have been identified, with the majority being associated with Temoneira (TEM), the Sulfhydryl Variable (SHV), and Cefotaxime hydrolyzing capabilities (CTX-M) (http://www.lahey.org/studies/webt.htm accessed on 15 July 2023). The majority of ESBLs found are either of the SHV or TEM types, and they are linked to nosocomial illnesses brought on by Gram-negative bacteria [3]. The presence of ESBLs carries enormous clinical importance. The ESBLs are frequently plasmid-encoded. Plasmids responsible for ESBL production frequently also carry resistance genes to other antibiotics. Although serious infections caused by ESBL-producing organisms are often treated with carbapenems, carbapenem-resistant isolates have recently been discovered [3]. In particular, the emergence of Enterobacterales resistant to third-generation cephalosporins and aztreonam, which is frequently associated with the expression of ESBLs, has caused antibiotic resistance among Gram-negative bacteria to rise rapidly over the past two decades [4]. According to the Clinical and Laboratory Standards Institute (CLSI) standards, regardless of the minimum inhibitory concentration (MIC) of a given cephalosporin, isolates with a positive phenotypic confirmatory test (combination disc tests/broth microdilution) should be reported as resistant to all cephalosporins (apart from the cephamycins, cefoxitin, and cefotetan) and aztreonam [5]. Furthermore, because the genes for ESBL synthesis are easily transferred by plasmids, many ESBL-producing Enterobacterales are also resistant to other antimicrobial drugs such as aminoglycosides, trimethoprim, and quinolones. This creates a severe dilemma in the treatment of ESBL-producing infections [6]. The incidence of ESBL-producing Enterobacteriaceae in hospitals and the general population varies greatly. Infections caused by Enterobacterales that produce ESBL are now of concern for a variety of reasons, including rising hospital expenditures, length of stay, and mortality rates [5].

Antibiotic resistance in *K. pneumoniae* is mainly driven by the acquisition of β-lactamases, such as extended-spectrum β-lactamases (ESBLs) and carbapenemases [7]. There is a greater risk of high mortality from infections caused by carbapenem-resistant, ESBL-producing, and multidrug-resistant Enterobacterales [8]. In fact, MDR *K. pneumoniae*, which produces carbapenemases, is regarded as a severe hazard to global health [1]. Although carbapenem antibiotics are the most effective therapeutic drug, due to their extensive usage, carbapenem-resistant *K. pneumoniae* (CRKP) is becoming more common throughout the world [9]. Several variables, such as the usage of antibiotics, horizontal gene transfer, and selective pressure in clinical settings, contribute to the emergence and spread of carbapenem resistance in *K. pneumoniae*. Colistin, in combination with tigecycline, rifampin, or carbapenem; fosfomycin plus colistin or amikacin; and double-carbapenem antibiotics (a combination of doripenem and ertapenem) are a few examples of combination antibiotics that are frequently used to treat CRKP [10]. Even though carbapenem-resistant Enterobacterales (CRE) and ESBL-producing strains frequently have similar genetic backgrounds, carry some of the same antibiotic-resistance determinants, and experience similar antibiotic pressures from beta-lactams, they are not always considered as a group when analyzing the epidemiology of multidrug-resistant Enterobacterales. Further explanation of the possible connections between the resistance profiles of CRE and ESBL-producing strains, as well as the role that these connections play in the spread of resistance, is required [11].

Furthermore, a review article on Gram-negative bacilli from the Gulf Cooperation Council (GCC) that produce β-lactamases revealed that the most prevalent β-lactamases are those with the carbapenemases genes OXA-48, NDM-1, and CTX-M-15 [12]. The rise of these resistant genes in numerous *K. pneumoniae* isolates in Saudi Arabia hospitals has also been demonstrated in several investigations [13,14,15]. The majority of those isolates were discovered in intensive care units (ICUs) from severely ill patients, and they had high fatality rates [12]. Even though there have not been many studies on CRKP isolates on the Arabian Peninsula, practically all of the GCC nations share similar ESBLs and carbapenemase-producing Enterobacterales, the bulk of which were isolated from nosocomial infections [16]. A study conducted in Saudi Arabia revealed a significant correlation between resistance determinants and the clonal types of *K. pneumoniae*. Specifically, all ST-152 isolates were found to be positive for NDM-1, while ST-199 isolates exhibited positivity for OXA-48. Additionally, both ST-709 and ST-199 isolates showed positivity for CTX-M-14 [17]. Furthermore, a study conducted in Iran reported the presence of *bla*_SHV_, *bla*_TEM_, *bla*_CTX-M-15_, *bla*_OXA-48_, *bla*_KPC_, and *bla*_NDM_ genes in 91.4%, 82.7%, 79.3%, 36.2%, 29.3%, and 6.9% of multidrug-resistant *K. pneumoniae* isolates, respectively [18]. Mahmoudi et al. demonstrated that out of 30 *K. pneumoniae* isolates, the frequency of *bla*_SHV_, *bla*_CTX-M-15_, and *bla*_TEM_ genes were 83% (*n* = 25), 70% (*n* = 21), and 57% (*n* = 17), respectively [19]. A study conducted in Iran reported that all the CRKP isolates were multidrug resistant. The study uncovered that 96% of CRKP isolates shared four common genes: *bla*_TEM_, *bla*_CTX−M-1_, *bla*_SHV_, and *bla*_CTX−M-15_, as reported by Abbasi and Ghaznavi-Rad [20]. Similarly, another study revealed that 93.6% of CRKP isolates shared three genes: *bla*_TEM_, *bla*_SHV_, and *bla*_CTX−M-15_, as reported by Solgi et al. [21]. These resistance genes are typically situated on mobile genetic elements, such as plasmids, which facilitate easy transferability within and between bacterial species. Moreover, studies conducted in the Middle East have demonstrated an increasing frequency of ESBL in *K. pneumoniae* over the past decade, as highlighted by Beigverdi [22]. Foreign travel was observed in approximately 23.3% of patients infected with CRE in the Gulf region [23]. Among the various travel destinations, India emerged as the primary country, followed by Africa and Pakistan. Notably, travel to and from Middle Eastern countries, including Saudi Arabia, has been identified as a source of OXA-48 carbapenemases in certain reports [23]. The initial case of OXA-48-producing carbapenem-resistant *Klebsiella pneumoniae* in the United States was recognized in a patient who had recently undergone hospitalization in Saudi Arabia [24].

Molecular typing techniques are helpful tools for managing and treating infections brought on by multi-drug-resistance organisms, showing the genetic links in nosocomial infection epidemics, and determining the most likely source of infection. The genetic relatedness of the CRKP isolates using molecular typing techniques such as pulsed-field gel electrophoresis (PFGE), multilocus sequence typing (MLST), or whole-genome sequencing (WGS) can help to recognize clonal relationships among the isolates and determine if certain genetic lineages are more prevalent. Molecular tools have limited resolution and a fairly longer turnaround time than whole-genome sequencing in guiding the treatment of MDR, XDR, and PDR infections. A molecular method called the enterobacterial repetitive intergenic consensus-polymerase chain reaction (ERIC-PCR) is also used to assess the genetic diversity among members of the Enterobacteriaceae family. The ERIC sequences are non-coding, conserved sections that are 126 bp long. The ERIC method can be applied to analyze genetic differences between bacterial strains. Bacteria contain ERIC sequences in different numbers and distributions. Examining the genetic similarities of bacterial isolates can be done quickly, accurately, and with high reliability using ERIC-PCR [25]. Strategies for control require ongoing monitoring of resistant strains. Understanding antibiotic resistance helps public health organizations, infection control committees, and antimicrobial stewardship programs make informed decisions about how to manage such resistant organisms [26].

Acknowledging the prevalence and dissemination of diverse antibiotic-resistance genes in *K. pneumoniae* both domestically and globally, as well as the scarcity of data in this specific geographic region, our objective was to examine the occurrence of CTX-M, SHV, and TEM genes in clinical isolates of CRKP from the Kingdom of Bahrain. To accomplish this, we employed molecular methods, including sequencing, for antibiotic resistance genes and also ERIC PCR to unravel the genetic relatedness among these isolates.

## 2. Materials and Methods

### 2.1. Bacterial Isolates and Hospital Setting

A total of twenty-four non-duplicate CRKP clinical isolates (December 2020 to June 2021) were included in this study. The bacterial strains were collected from Salmaniya Medical Complex, which is a multispecialty healthcare facility that provides secondary, tertiary, and emergency healthcare services in the Kingdom of Bahrain. It has approximately 1200 beds, with an average of 900 to 1000 people visiting the hospital each day, and it has more than 2000 doctors, nurses, and other workers. The bacterial strains were identified from the urine, blood, and endotracheal aspirate of the patients admitted to Salmaniya tertiary care hospital.

### 2.2. Bacterial Identification and Antimicrobial Susceptibility Testing

Bacterial species-level identification was established by using a mass spectrometry system (MALDI-TOF Bruker Daltonik GmbH, Bremen, Germany), and antibiotic susceptibility testing of isolates was performed with the VITEK-2 compact automated microbiological system (bioMerieux, Marcy L, Etoile, France). Only the isolates that were carbapenemase producers were included for further molecular analysis. The types of carbapenemases, the antibiotic resistance pattern, the genetic environment of *bla*_NDM_, integrons analysis, and molecular characterization of the plasmids of these isolates have already been published in our previous article [27].

### 2.3. DNA Isolation and Polymerase Chain Reaction-Based Amplification of Antibiotic-Resistant Genes

Fresh colonies from pure culture plates of *K. pneumoniae* clinical isolates were used to prepare whole-cell DNA. Each colony was suspended in 250 μL of nuclease-free water and incubated in a water bath at 95 °C for 15 min, followed by centrifugation at 12,000 rpm at 4 °C for 8 min. The supernatant was used as a template of DNA for polymerase chain reaction (PCR) on an Applied Biosystems GeneAmp PCR System 9700 (Foster City, CA, USA) with gene-specific primers as described earlier [28] to detect antimicrobial-resistant markers (*bla*_CTX-M_, *bla*_SHV_, and *bla*_TEM_). We have used bacterial strains having these resistant markers as positive control from strains available in Biorepository in the Department of Microbiology, Immunology, and Infectious Diseases, College of Medicine & Medical Sciences, Arabian Gulf University, Kingdom of Bahrain.

### 2.4. DNA Sequencing

At Geno Screen Lab, PCR-generated CTX-M fragments were sequenced (Campus Institut Pasteur de, La Calmette, France). Using the Clustal Omega tool (https://www.ebi.ac.uk/Tools/msa/clustalo/ accessed on 1 June 2023), the derived protein sequence was aligned with *bla*_CTX-M_ variants to verify the amino acid substitution in the query sequence for known variants. Additionally, online BLAST (versions 2.2.26) software (http://www.ncbi.nlm.nih.gov/BLAST/ accessed on 1 June 2023) was used to analyze the similarities between the amplified nucleotide sequence and the deduced protein sequences and was confirmed as *bla*_CTX-M-15_.

### 2.5. Molecular Genotyping

The genetic relatedness of ESBL-producing *K. pneumoniae* isolates was determined using enterobacterial repetitive intergenic consensus-PCR (ERIC-PCR) with specific primers as in our earlier published study [29]. Gel-electrophoresis was used to separate amplified PCR products in a 1.5% agarose gel containing ethidium bromide (0.5 µg/mL) with TAE running buffer. The Azure Biosystem C-200 Gel Documentation System was used to visualize gel images. PyElph version 1.4 software was used to analyze bands and create dendrogram clustering using the unweighted pair group method with arithmetic averages (UPGMA) [30].

## 3. Results

### 3.1. Antibiotic Resistance Genes Detection

All 24 *K. pneumoniae* isolates, which produce Extended Spectrum Beta-Lactamase (ESBL), were subjected to PCR assays to detect antibiotic-resistant determinants, including *bla*_CTX-M_, *bla*_SHV_, and *bla*_TEM_ genes. The results revealed a significant presence of these genes within the isolates: All isolates (100%) carried *bla*_SHV_ (24/24), 91.6% had *bla*_CTX-M_ (22/24), and 54.8% contained *bla*_TEM_ genes (13/24) (Table 1).

**Figure 1:**
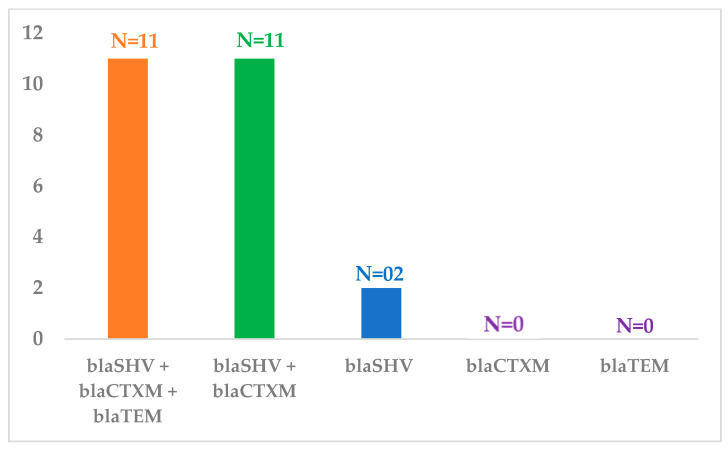
Frequency of *bla* genes present either in a single or in a combination in *Klebsiella pneumonia* isolates.

All 24 *K. pneumoniae* isolates, were found in various genes combinations, with 45.8% of isolates possessing all three genes (*bla*_SHV_ + *bla*_CTX-M_ + *bla*_TEM_, n = 11), 45.8% carrying a combination of *bla*_SHV_ + *bla*_CTX-M_ (n = 11), and a minority of 8.35% having only *bla*_SHV_ (n = 2). None of the isolates showed the presence of only (*bla*_CTX-M_ and *bla*_TEM_) genes (Figure 1).

### 3.2. Sequencing

Sequencing of the *bla*_CTX-M_ amplicons revealed the confirmation of *bla*_CTX-M-15_. Under the following accession numbers, these sequences have been added to the GenBank nucleotide database: OP807050, OP807051, OP807052, OP807053, OP807054, OP807055, OP807056, OP807057, OP807058, OP807059, OP807060, OP807061, OP807062, OP807063, OP807064, OP807065, OP807066, OP807067, OP807068, OP807069, OP807070, and OP807071 (Table 1), accessible at the National Center of Biotechnology Information website (http://www.ncbi.nlm.nih.gov accessed on 10 July 2023)

### 3.3. CRKP Isolates Clustering

**Figure 2:**
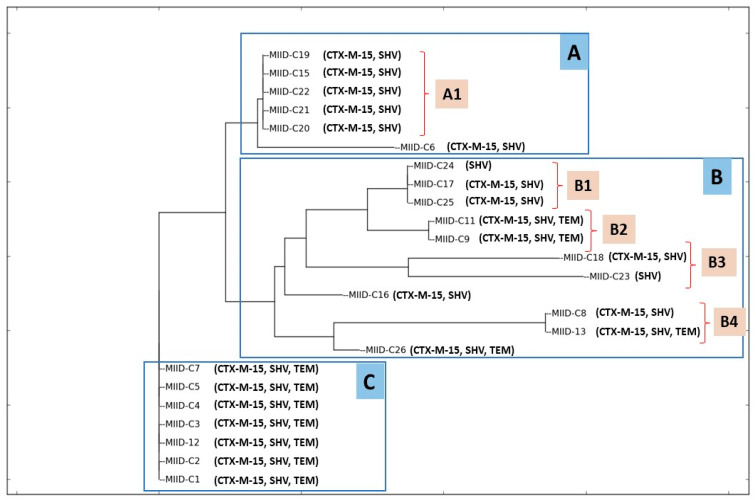
The dendrogram of CRKP isolates clustering based on ERIC patterns (A, B and C) along with the *bla* genes.

The ERIC-PCR profiles allowed differentiating 24 isolates into 3 ERIC clusters (A to C), where cluster A was further grouped into subgroup A1 and cluster B into four sub-clusters (B1, B2, B3, and B4) as shown in Figure 2. The isolates grouped within cluster A1 harbored an identical repertoire of resistance genes. Similarly, the isolates in cluster C possessed an identical complement of resistance determinants. Generally, the electrophoretic analysis of the PCR reaction products has revealed that the number of bands in particular electrophoretic paths ranged from 1 to 9. The sizes of the PCR products ranged from 400 bp to about 2000 bp.

### 3.4. ERIC Clusters and Their Antibiotic Resistance Pattern

**Figure 3:**
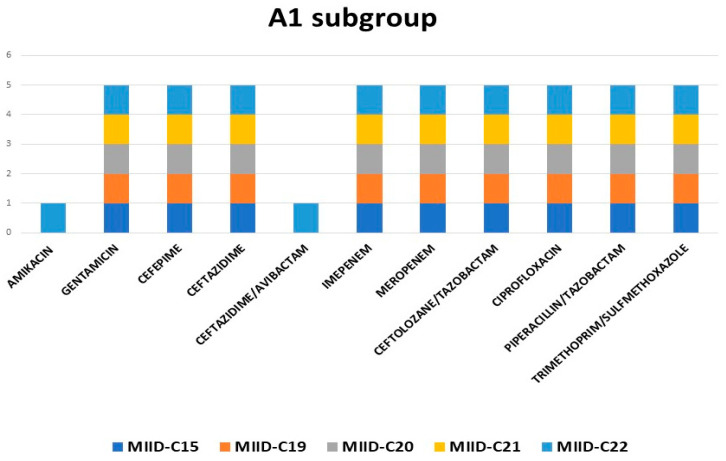
Antibiotic resistance pattern of subgroup A1.

Cluster A with subgroup A1 consisted of five isolates, and all these strains were isolated from blood. The CTX-M-15 and SHV genes were present in all five isolates. All of these isolates had the same pattern of antibiotic resistance, except for MIID-C22, which was resistant to both amikacin and ceftazidime-avibactam (Figure 3).

**Figure 4:**
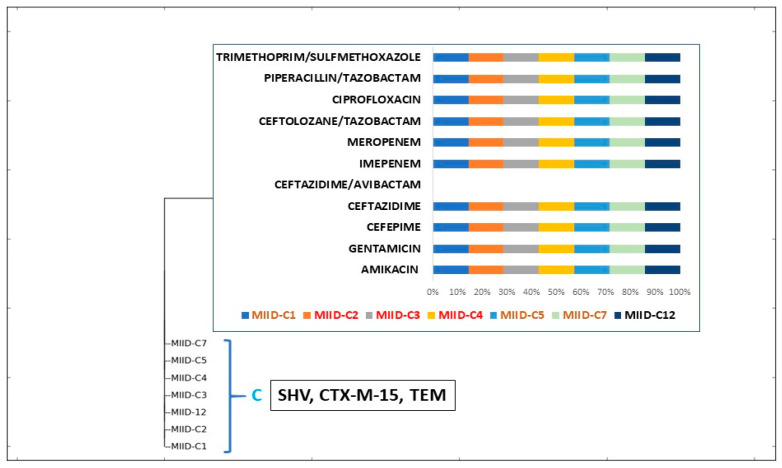
Antibiotic resistance pattern of cluster C isolates.

Cluster B was made up of four subclusters, B1 through B4. The resistance gene profiles within each subcluster varied significantly, according to ERIC analysis. Testing for antibiotic susceptibility also revealed variations in the antibiotic resistance patterns amongst the four subclusters. Cluster C consisted of seven isolates wherein MIID-C2, MIID-C3, MIID-C4 were isolated from blood, MIID-C1, MIID-C5, and MIID-C7 were from urine, and MIID-C12 was isolated from endotracheal aspirate of patients. The CTX-M-15, TEM, and SHV genes were present in all seven isolates. These isolates shared a common pattern of resistance to antibiotics. Except for ceftazidime-avibactam, all of these isolates were resistant to all known antibiotics (Figure 4).

## 4. Discussion

*Klebsiella pneumoniae* is generally resistant to multiple antibiotics and is considered a reservoir of antimicrobial resistance markers, and these can horizontally transfer to other Gram-negative pathogens [31]. The primary reason for therapy failure is the spread of CRKP, which also raises morbidity and mortality rates among hospital patients [32]. To effectively stop the spread of CRKP, accurate and rapid detection using molecular techniques, including PCR and/or whole genome sequencing, is crucial [16]. The primary factor causing carbapenem resistance in clinical isolates of *K. pneumoniae* is the emergence of several types of β-lactamases. It has been proven that *K. pneumoniae’s* resistance to broad-spectrum β-lactams and carbapenems is influenced by the production of ESBLs [33].

The simultaneous existence and evolution of antibiotic-resistant markers are considered to have the most worrying potential, as they may lead to the emergence of uncurable, disruptive *K. pneumoniae* infections [34]. In our study, the production of *bla*_SHV_, *bla*_CTX-M-15_, and *bla*_TEM_ combinations were found as two or three resistance genes in a single CRKP isolate. Moreover, it was observed that all the CRKP isolates demonstrated a significant presence of the SHV, CTX-M-15, and TEM genes (100%, 91.6%, and 45.8%), respectively. The SHV-type ESBL gene was the most prevalent among the isolates, followed by the CTX-M-type, which agrees with two previous reports [35,36]. Tijet et al. carried out a molecular characterization of KPC-producing Enterobacteriaceae submitted to the provincial reference laboratory in Ontario, Canada, due to the paucity of comprehensive reports of *Klebsiella pneumoniae* carbapenemase (KPC)-producing enterobacteria in that province. Their isolates harbored *bla*_KPC-2_ or *bla*_KPC-3_. The commonly detected gene was *bla*_TEM-1_, and occasionally *bla*_OXA-1_ and *bla*_CTX-M-15_ were detected. All *K. pneumoniae* isolates carried *bla*_SHV-11_ in the study [35]. Uz Zaman et al. reported the presence of CTX-M and SHV genes in all their isolates, with CTX-M-15 and SHV-1 types predominating among these extended-spectrum beta-lactamases (ESBLs). All isolates but one contained TEM-1 [36].

In Saudi Arabia, though carbapenem resistance remained rare among Enterobacterales, the first outbreak of CRKP was reported in 2010 [37]. In their study, a cluster of eight CRKPs were found in March 2010 in the adult intensive care unit. Two more CRKPs were discovered after a study of *K. pneumoniae* isolates from the previous six months. On performing PFGE, the bulk of the strains throughout the outbreak period were genetically similar or indistinguishable [37]. Later, the molecular basis of this resistance was found to be due to the involvement of carbapenemase enzyme (OXA-48) in combination with CTX-M-15 genes [36]. A study from India was conducted to ascertain the frequency and genetic makeup of *K. pneumoniae* strains that produce ESBL and carbapenemase isolated from intensive care units of a tertiary care hospital. Here, they reported the coexistence of the *bla* gene along with carbapenemase producers to be 69% and 50% of the SHV and TEM, respectively [38]. Previous Indian studies have documented the coexistence of carbapenemase producers (NDM) with the *bla* genes [39,40]. Similar international studies have revealed the presence of *bla* genes along with carbapenemase [41,42]. Carbapenemases-producing KP strains appear to frequently co-produce ESBL; as a result, they are resistant to both cephalosporins and carbapenems. Numerous researchers have noted this tendency, which shows that isolates that are resistant to multiple drugs frequently produce carbapenemase, which is also noticed in our study [42]. A study from China investigated the occurrence and spread of carbapenemase and extended-spectrum β-lactamase encoding genes co-existence in sporadic *K. pneumoniae* ST307 in pediatric patients from the Shenzhen Children’s Hospital, China, and reported the presence of SHV (92%) CTX-M (53%) among 36 CRKP isolates from pediatric patients [43]. A study from India reported *bla*_CTX-M_, *bla*_TEM_, and *bla*_SHV_ in coexistence with *bla*_NDM-1_ [44].

Globally, the CTX-M gene is linked to antibiotic resistance [36]. CTX-M sequencing of our isolates confirmed CTX-M-15 in our region. The same variant has been reported by Zaman et al. from Saudi Arabia as the most abundant ESBL gene detected in 47/71 (66.2%) CRKP isolates [17]. This is consistent with earlier studies [17,45,46] and suggests that it is endemic to this region. A study from China observed that among *bla_CTX-M_* producers, *bla_CTX-M-2_* was the most prevalent genotype (44/45, 97.8%), followed by *bla_CTX-M-3_* (20/45, 44.4%), *bla_CTX-M-1_* (18/45, 40%), *bla_CTX-M-9_* (17/45, 37.8%), and *bla_CTX-M-15_* (5/45, 11.1%) [47]. According to a study from Tanzania, ESBL genes were distributed as follows: 29/32 (90.6%) had bla_CTX-M-15_, two had bla_SHV-12_, and one had both bla_CTX-M-15_ and bla_SHV-12_. The percentage of hospital and community isolates with bla_CTX-M-15_ was 69% (20/29) and 31% (9/29), respectively. Only infections acquired in hospitals were found to have bla_SHV-12_ genotypes [48]. The rise of CRKP isolates, as well as co-existing *K. pneumoniae* strains of *bla*_CTX-M-1_ and *bla*_SHV_ in Lagos hospitals, were documented by a study from Nigeria [49]. In contrast to our findings, a study from Syria in 2015 reported the presence of bla_CTX-M-1_ (100%) as the most prevalent ESBL gene in *K. pneumoniae* [50]. Another study from Saudi Arabia reported *bla_CTX-M-1_* (60%) and *bla_CTX-M-9_*-like genes (40%) among *K. pneumoniae* [51].

Types of CTX-M ESBLs, especially CTX-M-15, are well known for spreading quickly among Enterobacteriaceae members all over the world [52]. A study by Blanco et al. reported that complicated UTI was strongly associated with ESBL-producing *E. coli* infections. In their study, CTX-M-15-producing *E. coli* showed ten different plusotypes; 65% were PT1 or PT4 and corresponded to ST131 [52]. Additionally, it has been proposed that the widespread use of ceftriaxone and cefotaxime may have contributed to the establishment and spread of CTX-M enzymes [53]. The emergence of carbapenemase-producing Gram-negatives is a worry because it is frequently linked with an outbreak of MDR isolates, which leads to restrictions on alternative treatments [54]. Although the carbapenemases had previously been reported in Saudi Arabia and Kuwait, no isolate was discovered to produce KPC, VIM, or IMP beta-lactamases. Since no carbapenemase activity or genes were found in the study isolates, there was a possibility that the resistance mechanism is unrelated to carbapenemase. These isolates generated genes of the CTX-M-15 type and most likely produced extended-spectrum beta-lactamases in connection with lower outer membrane permeability [54]. In the present study, findings of antibiotic susceptibility of *K. pneumoniae* isolates are in keeping with the reported MDR phenotype associated with detected antibiotic resistance markers of *bla*_SHV_, *bla*_CTX-M-15_, and *bla*_TEM_. However, the *K. pneumoniae* isolates revealed high resistance against multiple antibiotic groups, especially the carbapenem class. Resistance to carbapenem can be associated with other mechanisms, such as the production of AmpC and ESBL or modifications to the structure of the outer membrane [36,55].

A wide range of bacteria can be successfully typed using enterobacterial repetitive intergenic consensus (ERIC-PCR) sequences [56,57]. In our study, the ERIC-PCR profiles allowed differentiating CRKP isolates into 3 ERIC clusters. In a Danish investigation, a semi-automated rep-PCR typing method was utilized to identify the relationship between the strains associated with the outbreak and the ESBLs generated by local *K. pneumoniae* strains [58]. According to Kholy and Manakhly from Egypt, only 5 KPC-positive and two producers were genetically related when 27 CRKP isolates were analyzed using ERIC-PCR; the majority of the isolates were polyclonal [58]. ERIC-PCR can be used to look into the epidemiological relationships between MDR *K. pneumoniae* isolates and to determine whether any plausible outbreaks might exist. According to research by Kundu et al., no outbreaks or nosocomial clustering occurred throughout the planned sampling period based on ERIC-PCR typing results and their relationship to hospital wards and units of data [59].

In this study, the isolates of ERIC types A and C had similar antibiotic resistance patterns. The cluster A and C isolates were resistant to all the antibiotics except amikacin and ceftazidime/avibactam. Among this resistance pattern, an exception was MIID-C22, which was resistant to both amikacin and ceftazidime-avibactam. This could be due to the presence of other antibiotic resistance mechanisms that were not studied here. A study on CRKP colonization in an intensive care unit by Madni et al. identified five primary ERIC clusters. Except for ERIC cluster 2, where isolates showed two antibiograms, isolates from the same ERIC cluster typically had identical antibiograms [60]. According to research by Shen et al., CRKP isolates had lower resistance to the antibiotics trimethoprim/sulfamethoxazole (24.5%), ciprofloxacin (23.4%), gentamicin (22.3%), levofloxacin (17.0%), tobramycin (16.0%), and amikacin (14.9%). According to their findings, 76.6% of the isolates were resistant to ceftazidime/avibactam, while 21.3% were resistant to tigecycline [61].

The small sample size is the main limitation of this study. Due to this, it is difficult to generalize the findings to other carbapenemase-producing CRKP strains that harbor these *bla* genes. Hence, an extensive multi-center investigation is needed. Another limitation is that this study was restricted to carbapenemases in CRKP isolates, so the details of other molecular mechanisms were not known. Additionally, the inability to perform whole-genome sequencing and analysis would have revealed more about the genomic determinants of antibiotic resistance and virulence in these isolates.

To the best of our knowledge, this is the first publication that describes the *bla* genes and molecular typing of CRKP isolates from this region.

## 5. Conclusions

The study findings reveal the concomitant carriage of the SHV, CTX-M, and comparatively lower carriage of TEM genes in CRKP isolates. Sequencing of the CTX-M positive isolates confirmed CTX-M-15 in our region. CRKP infection is a significant clinical concern with a high mortality rate due to the difficult nature of treating CRKP and the lack of an optimum medication regimen. Our findings highlight the significance of routinely using molecular characterization assays in hospital laboratories for the accurate detection of antibiotic-resistance gene-carrying bacteria. It is crucial to report the presence of antibiotic-resistance genes along with regular antibiotic sensitivity reports, as this will aid the clinician in prescribing the appropriate antibiotics. This study has contributed to the understanding of antibiotic resistance genes and has provided valuable data on *bla* gene carriage in CRKP isolates. Studies with a larger sample size are required to objectively assess the trends and comprehend the dynamics of spread and efficient control strategies.

## Figures and Tables

**Table 1:** Distribution of *bla* genes in *Klebsiella pneumonia* isolates with their respective accession number.

Sample ID	Organism Name	Accession Number	*bla*_CTX-M_	*bla*_SHV_	*bla*_TEM_
C-1	*K. pneumoniae*	OP807050	Present	Present	Present
C-2	*K. pneumoniae*	OP807051	Present	Present	Present
C-3	*K. pneumoniae*	OP807052	Present	Present	Present
C-4	*K. pneumoniae*	OP807053	Present	Present	Present
C-5	*K. pneumoniae*	OP807054	Present	Present	Present
C-6	*K. pneumoniae*	OP807055	Present	Present	Present
C-7	*K. pneumoniae*	OP807056	Present	Present	NP
C-8	*K. pneumoniae*	OP807057	Present	Present	Present
C-9	*K. pneumoniae*	OP807058	Present	Present	Present
C-11	*K. pneumoniae*	OP807059	Present	Present	Present
C-12	*K. pneumoniae*	OP807060	Present	Present	Present
C-13	*K. pneumoniae*	OP807061	Present	Present	Present
C-15	*K. pneumoniae*	OP807062	Present	Present	Present
C-16	*K. pneumoniae*	OP807063	Present	Present	NP
C-17	*K. pneumoniae*	OP807064	Present	Present	NP
C-18	*K. pneumoniae*	OP807065	Present	Present	NP
C-19	*K. pneumoniae*	OP807066	Present	Present	NP
C-20	*K. pneumoniae*	OP807067	Present	Present	NP
C-21	*K. pneumoniae*	OP807068	Present	Present	NP
C-22	*K. pneumoniae*	OP807069	Present	Present	NP
C-23	*K. pneumoniae*	#	NP	Present	NP
C-24	*K. pneumoniae*	#	NP	Present	NP
C-25	*K. pneumoniae*	OP807070	Present	Present	NP
C-26	*K. pneumoniae*	OP807071	Present	Present	Present

NP: Corresponding *bla* gene not present in this isolate, **#** CTXM-15 sequence; not submitted to GenBank Database.

## Data Availability

All data used for analysis are presented as figures in this article.

## References

[1] Mil-Homens D., Martins M., Barbosa J., Serafim G., Sarmento M.J., Pires R.F., Rodrigues V., Bonifácio V.D.B., Pinto S.N. (2021). Carbapenem-Resistant *Klebsiella pneumoniae* Clinical Isolates: In Vivo Virulence Assessment in Galleria mellonella and Potential Therapeutics by Polycationic Oligoethyleneimine. Antibiotics.

[2] Eliopoulos G.M., Bush K. (2001). New β-lactamases in Gram-negative bacteria: Diversity and impact on the selection of antimicrobial therapy. Clin. Infect. Dis..

[3] Paterson D.L., Bonomo R.A. (2005). Extended-spectrum β-lactamases: A clinical update. Clin. Microbiol. Rev..

[4] Giriyapur R.S., Nandihal N.W., Patil A.B. (2011). Comparison of disc diffusion methods for the detection of extended-spectrum beta lactamase-producing Enterobacteriaceae. J. Lab. Physicians.

[5] Leverstein-van Hall M.A., Fluit A.C., Paauw A., Box A.T., Brisse S., Verhoef J. (2002). Evaluation of the Etest ESBL and the BD Phoenix, VITEK 1, and VITEK 2 automated instruments for detection of extended-spectrum beta-lactamases in multiresistant *Escherichia coli* and *Klebsiella* spp.. J. Clin. Microbiol..

[6] Tada D.G., Gandhi P.J., Patel K.N. (2012). A study on antibiotic related resistance in UTI patients: A comparison between community acquired and hospital acquired *E. coli*. Natl. J. Community Med..

[7] Ramadan R.A., Bedawy A.M., Negm E.M., Hassan T.H., Ibrahim D.A., ElSheikh S.M., Amer R.M. (2022). Carbapenem-resistant *Klebsiella pneumoniae* among patients with ventilator-associated pneumonia: Evaluation of antibiotic combinations and susceptibility to new antibiotics. Infect. Drug Resist..

[8] Weiner L.M., Webb A.K., Limbago B., Dudeck M.A., Patel J., Kallen A.J., Edwards J.R., Sievert D.M. (2016). Antimicrobial-resistant pathogens associated with healthcare-associated infections: Summary of data reported to the National Healthcare Safety Network at the Centers for Disease Control and Prevention, 2011–2014. Infect. Control Hosp. Epidemiol..

[9] Zhang R., Chan E.W., Zhou H., Chen S. (2017). Prevalence and genetic characteristics of carbapenem-resistant Enterobacteriaceae strains in China. Lancet. Infect. Dis..

[10] Jafari Z., Harati A.A., Haeili M., Kardan-Yamchi J., Jafari S., Jabalameli F., Meysamie A., Abdollahi A., Feizabadi M.M. (2019). Molecular Epidemiology and Drug Resistance Pattern of Carbapenem-Resistant *Klebsiella pneumoniae* Isolates from Iran. Microb. Drug Resist..

[11] Tian X., Sun S., Jia X., Zou H., Li S., Zhang L. (2018). Epidemiology of and risk factors for infection with extended-spectrum β-lactamase-producing carbapenem-resistant Enterobacteriaceae: Results of a double case–control study. Infect. Drug Resist..

[12] Zowawi H.M., Balkhy H.H., Walsh T.R., Paterson D.L. (2013). β-Lactamase production in key gram-negative pathogen isolates from the Arabian Peninsula. Clin. Microbiol. Rev..

[13] Alotaibi F. (2019). Carbapenem-Resistant Enterobacteriaceae: An update narrative review from Saudi Arabia. J. Infect. Public Health.

[14] Al-Abdely H., AlHababi R., Dada H.M., Roushdy H., Alanazi M.M., Alessa A.A., Gad N.M., Alasmari A.M., Radwan E.E., Al-Dughmani H. (2021). Molecular characterization of carbapenem-resistant Enterobacterales in thirteen tertiary care hospitals in Saudi Arabia. Ann. Saudi Med..

[15] Alghoribi M.F., Binkhamis K., Alswaji A.A., Alhijji A., Alsharidi A., Balkhy H.H., Doumith M., Somily A. (2020). Genomic analysis of the first KPC-producing *Klebsiella pneumoniae* isolated from a patient in Riyadh: A new public health concern in Saudi Arabia. J. Infect. Public Health.

[16] Al-Zahrani I.A., Alasiri B.A. (2018). The emergence of carbapenem-resistant *Klebsiella pneumoniae* isolates producing OXA-48 and NDM in the Southern (Asir) province, Saudi Arabia. Saudi Med. J..

[17] Alrodayyan M., Albladi M., Aldrees M., Siddique M.I., Aljohani S., Balkhy H.H. (2018). Clonal diversity and genetic profiling of antibiotic resistance among multidrug/carbapenem-resistant *Klebsiella pneumoniae* isolates from a tertiary care hospital in Saudi Arab. BMC Infect. Dis..

[18] Farhadi M., Ahanjan M., Goli H.R., Haghshenas M.R., Gholami M. (2021). High frequency of multidrug-resistant (MDR) *Klebsiella pneumoniae* harboring several β-lactamase and integron genes collected from several hospitals in the north of Iran. Ann. Clin. Microbiol. Antimicrob..

[19] Mahmoudi S., Pourakbari B., Rahbarimanesh A., Abdosalehi M.R., Ghadiri K., Mamishi S. (2019). An outbreak of ESBL-producing *Klebsiella pneumoniae* in an Iranian referral hospital: Epidemiology and molecular typing. Infect. Disord.-Drug Targets.

[20] Abbasi E., Ghaznavi-Rad E. (2023). High frequency of NDM-1 and OXA-48 carbapenemase genes among *Klebsiella pneumoniae* isolates in central Iran. BMC Microbiol..

[21] Solgi H., Badmasti F., Giske C.G., Aghamohammad S., Shahcheraghi F. (2018). Molecular epidemiology of NDM-1-and OXA-48-producing *Klebsiella pneumoniae* in an iranian hospital: Clonal dissemination of ST11 and ST893. J. Antimicrob. Chemother..

[22] Beigverdi R., Jabalameli L., Jabalameli F., Emaneini M. (2019). Prevalence of extended-spectrum β-lactamase-producing Klebsiella pneumoniae: First systematic review and meta-analysis from Iran. J. Glob. Antimicrob. Resist..

[23] Sonnevend A., Ghazawi A.A., Hashmey R., Jamal W., Rotimi V.O., Shibl A.M., Al-Jardani A., Al-Abri S.S., Tariq W.U., Weber S. (2015). Characterization of carbapenem-resistant Enterobacteriaceae with high rate of autochthonous transmission in the Arabian Peninsula. PLoS ONE.

[24] Mathers A.J., Hazen K.C., Carroll J., Yeh A.J., Cox H.L., Bonomo R.A., Sifri C.D. (2013). First clinical cases of OXA-48-producing carbapenem-resistant *Klebsiella pneumoniae* in the United States: The “menace” arrives in the new world. J. Clin. Microbiol..

[25] Abdelhamid S.M., Abd-Elaal H.M., Matareed M.O., Baraka K. (2020). Genotyping and Virulence Analysis of Drug Resistant Clinical *Klebsiella pneumoniae* isolates in Egypt. J. Pure Appl. Microbiol..

[26] Di Tella D., Tamburro M., Guerrizio G., Fanelli I., Sammarco M.L., Ripabelli G. (2019). Molecular epidemiological insights into colistin-resistant and carbapenemases-producing clinical *Klebsiella pneumoniae* isolates. Infect. Drug Resist..

[27] Shahid M., Ahmad N., Saeed N.K., Shadab M., Joji R.M., Al-Mahmeed A., Bindayna K.M., Tabbara K.S., Dar F.K. (2022). Clinical carbapenem-resistant *Klebsiella pneumoniae* isolates simultaneously harboring *bla*NDM-1, *bla*OXA types and qnrS genes from the Kingdom of Bahrain: Resistance profile and genetic environment. Front. Cell. Infect. Microbiol..

[28] Shahid M. (2010). Citrobacter spp. simultaneously harboring *bla*CTX-M, *bla*TEM, *bla*SHV, *bla*ampC, and insertion sequences IS26 and orf513: An evolutionary phenomenon of recent concern for antibiotic resistance. J. Clin. Microbiol..

[29] Shahid M., Malik A., Akram M., Agrawal L.M., Khan A.U., Agrawal M. (2008). Prevalent phenotypes and antibiotic resistance in *Escherichia coli* and *Klebsiella pneumoniae* at an Indian tertiary care hospital: Plasmid-mediated cefoxitin resistance. Int. J. Infect. Dis. IJID Off. Publ. Int. Soc. Infect. Dis..

[30] Pavel A.B., Vasile C.I. (2012). PyElph—A software tool for gel images analysis and phylogenetics. BMC Bioinform..

[31] Chung H., Karkey A., Pham Thanh D., Boinett C.J., Cain A.K., Ellington M., Baker K.S., Dongol S., Thompson C., Harris S.R. (2015). A high-resolution genomic analysis of multidrug-resistant hospital outbreaks of Klebsiella pneumoniae. EMBO Mol. Med..

[32] Pavelkovich A., Balode A., Edquist P., Egorova S., Ivanova M., Kaftyreva L., Konovalenko I., Kõljalg S., Lillo J., Lipskaya L. (2014). Detection of carbapenemase-producing enterobacteriaceae in the baltic countries and st. Petersburg area. BioMed Res. Int..

[33] Nordmann P., Cuzon G., Naas T. (2009). The real threat of *Klebsiella pneumoniae* carbapenemase-producing bacteria. Lancet. Infect. Dis..

[34] Alraddadi B.M., Heaphy E.L.G., Aljishi Y., Ahmed W., Eljaaly K., Al-Turkistani H.H., Alshukairi A.N., Qutub M.O., Alodini K., Alosaimi R. (2022). Molecular Epidemiology and Outcome of Carbapenem-Resistant Enterobacterales in Saudi Arabia. BMC Infect. Dis..

[35] Tijet N., Sheth P.M., Lastovetska O., Chung C., Patel S.N., Melano R.G. (2014). Molecular characterization of *Klebsiella pneumoniae* carbapenemase (KPC)-producing Enterobacteriaceae in Ontario, Canada, 2008–2011. PLoS ONE.

[36] Uz Zaman T., Aldrees M., Al Johani S.M., Alrodayyan M., Aldughashem F.A., Balkhy H.H. (2014). Multi-drug carbapenem-resistant *Klebsiella pneumoniae* infection carrying the OXA-48 gene and showing variations in outer membrane protein 36 causing an outbreak in a tertiary care hospital in Riyadh, Saudi Arabia. Int. J. Infect. Dis. IJID Off. Publ. Int. Soc. Infect. Dis..

[37] Balkhy H.H., El-Saed A., Al Johani S.M., Francis C., Al-Qahtani A.A., Al-Ahdal M.N., Altayeb H.T., Arabi Y., Alothman A., Sallah M. (2012). The epidemiology of the first described carbapenem-resistant *Klebsiella pneumoniae* outbreak in a tertiary care hospital in Saudi Arabia: How far do we go?. Eur. J. Clin. Microbiol. Infect. Dis. Off. Publ. Eur. Soc. Clin. Microbiol..

[38] Bhaskar B.H., Mulki S.S., Joshi S., Adhikary R., Venkatesh B.M. (2019). Molecular Characterization of Extended Spectrum β-lactamase and Carbapenemase Producing *Klebsiella pneumoniae* from a Tertiary Care Hospital. Indian. J. Crit. Care Med. Peer-Rev. Off. Publ. Indian. Soc. Crit. Care Med..

[39] Ahmad N., Khalid S., Ali S.M., Khan A.U. (2018). Occurrence of *bla*(NDM) Variants among Enterobacteriaceae from a Neonatal Intensive Care Unit in a Northern India Hospital. Front. Microbiol..

[40] Ahmad N., Ali S.M., Khan A.U. (2020). Co-existence of *bla*NDM-1 and *bla*VIM-1 producing Moellerella wisconsensis in NICU of North Indian Hospital. J. Infect. Dev. Ctries..

[41] Jeremiah S.S., Balaji V., Anandan S., Sahni R.D. (2014). A possible alternative to the error prone modified Hodge test to correctly identify the carbapenemase producing Gram-negative bacteria. Indian. J. Med. Microbiol..

[42] Wang X., Chen G., Wu X., Wang L., Cai J., Chan E.W., Chen S., Zhang R. (2015). Increased prevalence of carbapenem resistant Enterobacteriaceae in hospital setting due to cross-species transmission of the *bla*NDM-1 element and clonal spread of progenitor resistant strains. Front. Microbiol..

[43] Patil S., Chen H., Guo C., Zhang X., Ren P.G., Francisco N.M., Wen F. (2021). Emergence of *Klebsiella pneumoniae* ST307 Co-Producing CTX-M with SHV and KPC from Paediatric Patients at Shenzhen Children’s Hospital, China. Infect. Drug Resist..

[44] Ahmad N., Ali S.M., Khan A.U. (2017). First reported New Delhi metallo-β-lactamase-1-producing Cedecea lapagei. Int. J. Antimicrob. Agents.

[45] Lee M.Y., Ko K.S., Kang C.-I., Chung D.R., Peck K.R., Song J.-H. (2011). High prevalence of CTX-M-15-producing *Klebsiella pneumoniae* isolates in Asian countries: Diverse clones and clonal dissemination. Int. J. Antimicrob. Agents.

[46] Al-Marzooq F., Mohd Yusof M.Y., Tay S.T. (2015). Molecular analysis of antibiotic resistance determinants and plasmids in Malaysian isolates of multidrug resistant Klebsiella pneumoniae. PLoS ONE.

[47] Wang S., Dong H., Wang M., Ma W., Cheng Y., Zhou J., Cheng Y., Xu H., Yu X. (2022). Molecular Epidemiology of Carbapenem-Resistant *Klebsiella pneumoniae* in a Tertiary Hospital in Northern China. Can. J. Infect. Dis. Med. Microbiol..

[48] Manyahi J., Moyo S.J., Tellevik M.G., Ndugulile F., Urassa W., Blomberg B., Langeland N. (2017). Detection of CTX-M-15 beta-lactamases in Enterobacteriaceae causing hospital- and community-acquired urinary tract infections as early as 2004, in Dar es Salaam, Tanzania. BMC Infect. Dis..

[49] Akinyemi K.O., Abegunrin R.O., Iwalokun B.A., Fakorede C.O., Makarewicz O., Neubauer H., Pletz M.W., Wareth G. (2021). The Emergence of *Klebsiella pneumoniae* with Reduced Susceptibility against Third Generation Cephalosporins and Carbapenems in Lagos Hospitals, Nigeria. Antibiotics.

[50] AL-Subol I., Youssef N. (2015). Prevalence of CTX-M, TEM and SHV Beta-lactamases in Clinical Isolates of *Escherichia coli* and *Klebsiella pneumoniae* Isolated from Aleppo University Hospitals, Aleppo, Syria. Arch. Clin. Infect. Dis..

[51] Al-Agamy M.H.M., Shibl A.M., Tawfik A.F. (2009). Prevalence and molecular characterization of extended-spectrum beta-lactamase-producing *Klebsiella pneumoniae* in Riyadh, Saudi Arabia. Ann. Saudi Med..

[52] Blanco V.M., Maya J.J., Correa A., Perenguez M., Munoz J.S., Motoa G., Pallares C.J., Rosso F., Matta L., Celis Y. (2016). Prevalence and risk factors for extended-spectrum β-lactamase-producing *Escherichia coli* causing community-onset urinary tract infections in Colombia. Enfermedades Infecc. Y Microbiol. Clin..

[53] Wang H., Kelkar S., Wu W., Chen M., Quinn J.P. (2003). Clinical isolates of Enterobacteriaceae producing extended-spectrum β-lactamases: Prevalence of CTX-M-3 at a hospital in China. Antimicrob. Agents Chemother..

[54] Zowawi H.M., Sartor A.L., Balkhy H.H., Walsh T.R., Al Johani S.M., AlJindan R.Y., Alfaresi M., Ibrahim E., Al-Jardani A., Al-Abri S. (2014). Molecular Characterization of Carbapenemase-Producing *Escherichia coli* and *Klebsiella pneumoniae* in the Countries of the Gulf Cooperation Council: Dominance of OXA-48 and NDM Producers. Antimicrob. Agents Chemother..

[55] Memish Z.A., Assiri A., Almasri M., Roshdy H., Hathout H., Kaase M., Gatermann S.G., Yezli S. (2015). Molecular Characterization of Carbapenemase Production among Gram-Negative Bacteria in Saudi Arabia. Microb. Drug Resist..

[56] Nielsen J.B., Skov M.N., Jørgensen R.L., Heltberg O., Hansen D.S., Schønning K. (2011). Identification of CTX-M15-, SHV-28-producing *Klebsiella pneumoniae* ST15 as an epidemic clone in the Copenhagen area using a semi-automated Rep-PCR typing assay. Eur. J. Clin. Microbiol. Infect. Dis..

[57] Ahmad N., Ali S.M., Khan A.U. (2018). Detection of New Delhi Metallo-β-Lactamase Variants NDM-4, NDM-5, and NDM-7 in Enterobacter aerogenes Isolated from a Neonatal Intensive Care Unit of a North India Hospital: A First Report. Microb. Drug Resist..

[58] Kholy A.E., Manakhly A.E. (2018). 2300. Molecular Epidemiology of Carbapenem-Resistant *Klebsiella pneumoniae* (CRKP) Causing Central Line Associated Blood Stream Infections (CLABSI) in Three ICU Units in Egypt. Open Forum Infect. Dis..

[59] Kundu J., Kansal S., Rathore S., Kaundal M., Angrup A., Biswal M., Walia K., Ray P. (2022). Evaluation of ERIC-PCR and MALDI-TOF as typing tools for multidrug resistant *Klebsiella pneumoniae* clinical isolates from a tertiary care center in India. PLoS ONE.

[60] Madni O., Amoako D.G., Abia A.L.K., Rout J., Essack S.Y. (2021). Genomic Investigation of Carbapenem-Resistant *Klebsiella pneumonia* Colonization in an Intensive Care Unit in South Africa. Genes.

[61] Shen M., Chen X., He J., Xiong L., Tian R., Yang G., Zha H., Wu K. (2023). Antimicrobial Resistance Patterns, Sequence Types, Virulence and Carbapenemase Genes of Carbapenem-Resistant *Klebsiella pneumoniae* Clinical Isolates from a Tertiary Care Teaching Hospital in Zunyi, China. Infect. Drug Resist..

